# The epistemology of death: psychological autopsy, artificial intelligence, and forensic decision-making in equivocal deaths

**DOI:** 10.3389/fpsyg.2026.1900172

**Published:** 2026-07-09

**Authors:** Kenan Kaya, Ziyaettin Erdem, Özlem Akpınar, Derya Kaya

**Affiliations:** 1 Department of Forensic Medicine, Faculty of Medicine, Cukurova University, Adana, Türkiye; 2 Council of Forensic Medicine, Eskişehir Branch Directorate, Eskişehir, Türkiye; 3 Department of Psychiatry, Adana Dr. Ekrem Tok Mental Health and Neurological Diseases Hospital, Adana, Türkiye

**Keywords:** artificial intelligence, equivocal deaths, forensic psychology, psychological autopsy, suicide research

## Abstract

Traditional post-mortem examinations primarily focus on determining the biological cause of death (causa mortis). However, in equivocal death cases, understanding the intention underlying death may be as important as identifying its physiological mechanism. Psychological autopsy has emerged as a retrospective and interdisciplinary approach aimed at reconstructing the decedent’s mental state, behavioral patterns, and psychosocial circumstances preceding death. This review provides a critical and integrative examination of psychological autopsy by synthesizing its classical theoretical foundations with recent developments in artificial intelligence and digital forensic technologies, while examining its methodological, socio-psychological, temporal, technological, epistemological, and legal dimensions. Rather than treating psychological autopsy as a definitive investigative technique, the review examines its epistemological assumptions, methodological limitations, and evidentiary implications. Particular attention is given to its continuing practical relevance within the Turkish legal system despite the absence of explicit statutory regulation. The review argues that psychological autopsy is best understood as a structured interpretive framework grounded in probabilistic reasoning, data triangulation, and contextual analysis. Furthermore, advances in machine learning and natural language processing have introduced new forms of computational inference into post-mortem psychological reconstruction, expanding both methodological possibilities and the ethical and legal challenges associated with forensic decision-making. While previous studies have largely examined psychological autopsy from clinical, forensic, or legal perspectives separately, limited attention has been given to their integration within the context of artificial intelligence and digital transformation. The study ultimately highlights the need for greater methodological standardization, interdisciplinary integration, and ethical oversight in the evolving landscape of psychological autopsy practice.

## 1 Introduction

Death has traditionally been conceptualized within forensic science as a biological event defined through observable physiological processes. Established post-mortem techniques enable the identification of bodily changes, including rigor mortis, livor mortis, and decomposition, thereby facilitating the determination of the cause and manner of death. However, this biologically grounded approach reveals a fundamental limitation: while it may explain how death occurred, it frequently remains insufficient to explain why death occurred under specific circumstances.

This limitation becomes particularly significant in cases classified as equivocal deaths, in which the distinction between suicide, accident, and homicide cannot be readily established. In such cases, the determination of intent carries substantial legal, ethical, and social implications. The final classification of death may carry important legal, financial, and social consequences. Against this background, the need for interpretive approaches capable of addressing the psychological dimensions of death becomes increasingly apparent.

The emergence of psychological autopsy represents an attempt to address this interpretive gap. First systematically conceptualized by Shneidman (1981), psychological autopsy seeks to reconstruct the internal experiences of the deceased through the examination of behavioral patterns, emotional states, interpersonal relationships, and environmental stressors preceding death.

Despite its conceptual significance, psychological autopsy remains methodologically contested. Its retrospective nature necessitates reliance on indirect sources of information, including interviews with relatives, documentary materials, and behavioral traces. As emphasized by Cavanagh et al. (2003), such reconstructions are inherently probabilistic and remain vulnerable to various forms of bias. These limitations have generated ongoing debates regarding the reliability, validity, and evidentiary value of psychological autopsy findings within both forensic and academic contexts.

Recent technological developments have further complicated this issue. The digitalization of everyday life has generated extensive behavioral data, commonly described as “digital footprints.” These include social media interactions, search histories, and personal communications, collectively providing a continuous and temporally structured record of individual behavior. The increasing availability of such data has expanded the methodological possibilities of psychological autopsy and forensic investigation. Nevertheless, the incorporation of digital information into forensic analysis also introduces new challenges, including algorithmic bias, interpretive overreach, and ethical concerns related to privacy.

Another critical dimension concerns the legal applicability of psychological autopsy. Although the method has been recognized in various jurisdictions as a form of expert analysis, its evidentiary status remains ambiguous. In Turkey, judicial practice, particularly decisions of the Yargıtay, suggests an implicit recognition of the importance of examining the mental state of the deceased. Nevertheless, the absence of explicit legal codification has contributed to inconsistencies in both application and interpretation.

In light of these considerations, a clear research gap emerges. Existing literature has generally approached psychological autopsy as either a clinical instrument, a forensic technique, or a legal mechanism, while only rarely integrating these perspectives within the context of ongoing technological transformation.

Accordingly, this review aims to provide a critical and integrative examination of psychological autopsy. Specifically, it seeks to: (1) evaluate its methodological foundations and limitations; (2) analyze its evidentiary role in equivocal death cases; (3) assess the implications of artificial intelligence and digital transformation; and (4) examine its applicability within the Turkish legal system.

Rather than presenting psychological autopsy as a definitive solution, this review conceptualizes it as a structured interpretive framework operating under conditions of uncertainty.

## 2 Methodological considerations

Psychological autopsy lacks a universally accepted standardized protocol. Although structured approaches have been proposed (Jobes et al., 1991), they primarily function as heuristic models rather than strict scientific procedures. The absence of standardization creates considerable variability across forensic institutions, particularly with respect to the collection, weighting, and interpretation of data. Consequently, methodological outcomes frequently depend on the expertise and interpretive judgment of the investigator rather than on fully reproducible procedural standards.

Triangulation constitutes the central methodological principle of psychological autopsy, integrating interviews, medical documentation, financial records, and behavioral indicators.

Each source contributes a distinct epistemic dimension: interviews provide subjective narrative reconstructions, medical records offer clinical histories, and behavioral and financial data provide objective yet indirect indicators of mental state. Despite this multiplicity of sources, subjectivity cannot be entirely eliminated, as interpretation remains unavoidable in the reconstruction of mental states (Cavanagh et al., 2003).

Another significant methodological concern involves retrospective reconstruction bias. Informants may unintentionally reinterpret the behavior of the deceased in a manner consistent with post-event knowledge, thereby compromising temporal accuracy.

Furthermore, selection bias may arise when only certain informants are available or willing to participate, potentially distorting the evidentiary foundation of the investigation. Collectively, these limitations demonstrate that psychological autopsy operates not as a deterministic diagnostic procedure but rather as an inferential framework grounded in probabilistic reasoning.

In addition to the traditional limitations of psychological autopsy, the integration of digital data introduces distinct methodological challenges. Whereas retrospective reconstruction, recall bias, and informant selection are inherent to conventional psychological autopsy, digital approaches raise concerns related to algorithmic bias, data representativeness, platform-specific behaviors, and the interpretability of computational outputs. These challenges require separate methodological consideration, as they originate from different epistemic sources and may influence forensic conclusions in different ways.

## 3 Socio-psychological dynamics

The interpretation of suicide is deeply embedded within social structures. Since the pioneering work of Durkheim (1897), suicide has been conceptualized as a socially influenced phenomenon rather than solely as an expression of individual pathology.

Durkheim’s typology—egoistic, altruistic, anomic, and fatalistic suicide—continues to provide a valuable framework for understanding how social integration and social regulation influence self-destructive behavior.

In contemporary contexts, socio-psychological dynamics extend beyond traditional social relationships to encompass digital and media environments. The Werther Effect (Phillips, 1974) demonstrates that sensationalized media reporting may contribute to imitative behavior, whereas the Papageno Effect (Niederkrotenthaler et al., 2010) highlights the protective function of narratives emphasizing coping strategies and psychological resilience. These findings suggest that the dissemination of information itself constitutes a significant factor in suicidal behavior, thereby influencing the interpretive framework of psychological autopsy.

Furthermore, interpersonal network structures, including family cohesion, peer relationships, and workplace-related stressors, substantially influence the psychological trajectories of individuals prior to death. Social isolation, perceived burdensomeness, and diminished belongingness have consistently been identified as critical risk factors in empirical research. Within the framework of psychological autopsy, these variables are reconstructed retrospectively; however, their interpretation is frequently mediated by informant bias and culturally shaped perceptions of suicide.

Accordingly, socio-psychological analysis within psychological autopsy should not be regarded as merely descriptive but rather as fundamentally interpretive, requiring the integration of cultural norms, communication environments, and relational dynamics.

## 4 Temporal dynamics

A critical phase often precedes suicide, characterized by behavioral and emotional changes that may be externally misinterpreted (Hawton and Appleby, 1988). This period, frequently conceptualized as the “presuicidal syndrome,” may involve cognitive constriction, heightened hopelessness, social withdrawal, and marked affective instability.

Psychological autopsy seeks to reconstruct this phase; however, its retrospective nature inherently limits temporal accuracy. Temporal reconstruction is particularly challenging because behavioral indicators are often nonlinear and highly context-dependent. For instance, an apparent improvement in mood may paradoxically coincide with an increased risk of suicide, particularly when individuals experience a sense of resolution following the decision to die by suicide.

Another key temporal issue concerns the variability of warning signs across individuals. While some cases demonstrate a gradual deterioration over weeks or months, others involve rapid escalation within hours or days. This heterogeneity complicates the development of predictive models and limits the generalizability of standardized temporal indicators.

In addition, memory decay among informants further reduces temporal precision. Events occurring closer to the time of death are typically recalled with greater clarity, whereas earlier behavioral changes may be reconstructed less accurately. Consequently, temporal reconstruction in psychological autopsy remains inherently probabilistic and should be interpreted within an acknowledged margin of uncertainty.

## 5 Artificial intelligence and digital autopsy

Digital traces provide continuous behavioral datasets that substantially expand the scope of forensic analysis. Computational methods, including natural language processing (NLP), sentiment analysis, and machine learning classification models, may assist in identifying emotional patterns, linguistic markers of depression, and behavioral anomalies preceding death (Buda et al., 2020).

Social media platforms, messaging applications, and search engine histories collectively constitute what may be described as a “digital behavioral archive.” Such an archive enables the longitudinal reconstruction of emotional states in ways that were previously unavailable to forensic investigators.

However, these systems remain fundamentally probabilistic and are highly dependent on training data, feature selection, and model architecture. Consequently, they are vulnerable to algorithmic bias, particularly when datasets are not culturally or linguistically representative. Moreover, excessive reliance on computational outputs may result in interpretive overreach, whereby statistical correlations are misinterpreted as causal psychological explanations.

Empirical evidence regarding the reliability of these computational approaches remains mixed. While some studies suggest that machine learning models may enhance the identification of behavioral risk patterns and facilitate large-scale data analysis, others have highlighted concerns regarding their generalizability, explainability, and susceptibility to context-dependent misclassification. These inconsistencies underscore the need for cautious interpretation and continued human oversight in forensic applications.

From an ethical perspective, digital autopsy raises significant concerns regarding postmortem data ownership, privacy, and consent. The extraction and analysis of personal digital content after death challenge traditional conceptions of privacy. Therefore, artificial intelligence should be conceptualized as an assistive tool rather than a substitute for expert forensic judgment.

## 6 Epistemological and ethical challenges

Psychological autopsy is constrained by recall bias, ethical concerns, and cultural variability. Informant narratives are shaped by grief, guilt, and social expectations, all of which may distort factual reconstruction (Hawton and Appleby, 1988).

From an epistemological perspective, the method operates within a probabilistic interpretive framework rather than a strictly positivist scientific model. Psychological states cannot be directly observed and must therefore be inferred from indirect behavioral and contextual indicators. This limitation raises important concerns regarding validity, reliability, and falsifiability.

Cultural variability further complicates interpretation, as expressions of distress and attitudes toward suicide differ substantially across societies. Consequently, identical behavioral indicators may carry different meanings depending on the cultural context in which they occur.

From an ethical standpoint, post-mortem privacy constitutes a central concern. The analysis of digital communications, diaries, and personal records raises important questions regarding informational rights after death. In addition, interviews with bereaved relatives may result in secondary psychological harm, thereby necessitating careful ethical oversight.

## 7 Legal status in Turkey

In Turkey, psychological autopsy is not codified as an independent forensic discipline but is generally utilized within the broader category of expert opinion. This has resulted in a dual structure characterized by both flexibility and legal ambiguity.

Judicial decisions of the Yargıtay demonstrate increasing recognition of the relevance of psychological evaluation in equivocal death cases (Yargıtay 12th Criminal Chamber, 2015). In certain cases, the failure to adequately examine the mental state of the deceased has been regarded as a procedural deficiency, thereby indirectly reinforcing the relevance of psychological autopsy within judicial reasoning.

However, no standardized national protocol currently exists regarding data collection, methodological structure, or evidentiary weighting. As a result, considerable variability may arise across cases and among experts.

Furthermore, the increasing role of digital evidence introduces additional legal challenges. Issues such as data admissibility, post-mortem privacy rights, and digital consent remain insufficiently regulated under Turkish law. The absence of clear legislative guidance contributes to uncertainty in both forensic practice and judicial interpretation.

Accordingly, the development of a formal regulatory framework may facilitate greater standardization of practice, enhance methodological reliability, and promote consistency in judicial decision-making.

## 8 Conclusion

Psychological autopsy occupies a unique position within forensic science, operating at the intersection of empirical observation and interpretive reconstruction. Unlike traditional forensic methods that rely primarily on physical evidence, psychological autopsy seeks to reconstruct the internal mental state of a deceased individual—an inherently unobservable phenomenon.

This study demonstrates that its primary value does not lie in producing definitive conclusions but rather in structuring uncertainty in a systematic and methodologically transparent manner. In equivocal death investigations, this structured uncertainty becomes particularly significant, as legal systems often require probabilistic reasoning in the absence of direct evidence. Psychological autopsy therefore functions as an epistemic bridge between behavioral science and legal decision-making.

The integration of artificial intelligence and digital data represents a substantial methodological transformation. Digital footprints provide unprecedented insight into behavioral trajectories; however, this expansion of data does not eliminate interpretive ambiguity. Instead, it redistributes uncertainty into algorithmic systems, where bias may be embedded in data selection, labeling practices, and model architecture. As noted in the forensic psychiatry literature, computational inference cannot replace clinical judgment but may serve as a complementary analytic layer (Buda et al., 2020). In this respect, artificial intelligence simultaneously complements and challenges traditional psychological autopsy. It complements conventional approaches by expanding access to longitudinal behavioral data and pattern recognition capabilities, yet it challenges them by introducing new forms of opacity, uncertainty, and dependence on computational assumptions.

From an ethical perspective, psychological autopsy raises fundamental questions regarding post-mortem privacy and digital identity. The reconstruction of psychological states through digital records challenges traditional legal and moral boundaries surrounding informational autonomy after death. This concern becomes more pronounced in contexts where legal frameworks do not clearly define the status of digital remains. Consequently, ethical governance must extend beyond consent-based models toward post-mortem data stewardship frameworks (Edwards and Harbinja, 2013).

Within the Turkish legal system, the method is partially recognized but not formally regulated. Judicial practice indicates an increasing reliance on psychological interpretation in equivocal death cases, particularly through expert reports and indirect behavioral evidence.

However, the absence of standardized procedural guidelines leads to variability in forensic practice and evidentiary evaluation, thereby reducing reproducibility and potentially affecting judicial certainty (Yargıtay 12th Criminal Chamber, 2015).

From an epistemological perspective, psychological autopsy reflects the broader limitations of forensic science in addressing mental phenomena. As emphasized in classical sociological and suicidological literature, human behavior cannot be fully reduced to observable physical traces (Durkheim, 1897; Hawton and Appleby, 1988). Instead, its interpretation requires frameworks that integrate social, psychological, and contextual dimensions. This position situates psychological autopsy within a probabilistic rather than a deterministic scientific paradigm.

The future development of the field may depend on three interconnected dimensions. First, methodological standardization is required to reduce variability in data collection and interpretation. Second, interdisciplinary integration—particularly among forensic psychology, data science, and law—must be strengthened to ensure analytical coherence. Third, ethical and legal regulation must evolve in parallel with technological capabilities, particularly with respect to digital evidence and post-mortem data rights.

Ultimately, psychological autopsy should be understood not as a standalone diagnostic tool but as part of a broader evidentiary ecosystem. Its strength lies not in certainty but in its ability to organize fragmented and indirect information into a coherent interpretive structure. In this sense, it reflects not only the ambiguity of death itself but also the epistemological limits of forensic certainty in the analysis of complex human phenomena.

This study contributes to the literature by reframing psychological autopsy as a hybrid epistemic system operating at the intersection of forensic inference and computational augmentation.

## References

[ref1] Buda M. Brent D. A. Melhem N. M. (2020). Machine learning approaches in forensic psychiatry: opportunities and risks for suicide prediction and postmortem analysis. J. Forensic Sci. 65, 1702–1711. doi: 10.1111/1556-4029.14420

[ref2] Cavanagh J. T. O. Carson A. J. Sharpe M. Lawrie S. M. (2003). Psychological autopsy studies of suicide: a systematic review. Psychol. Med. 33, 395–405. doi: 10.1017/S0033291702006943, 12701661

[ref3] Durkheim É. (1897). Suicide: A Study in Sociology. Detroit: Free Press.

[ref4] Edwards L. Harbinja E. (2013). Protecting post-mortem privacy: a legal and ethical framework. Int. Rev. Law Comput. Technol. 27, 94–108. doi: 10.1080/13600869.2013.764836

[ref5] Hawton K. Appleby L. (1988). Suicide and attempted suicide in young people. Br. J. Psychiatry 153, 111–120.3066430

[ref6] Jobes D. A. Berman A. L. Josselson A. R. (1991). The impact of psychological autopsies on medical examiners’ determination of manner of death. J. Forensic Sci. 31, 177–189. doi: 10.1520/jfs11870j 3944561

[ref7] Niederkrotenthaler T. Voracek M. Herberth A. Till B. Strauss M. Etzersdorfer E. . (2010). Role of media reports in completed and prevented suicide: Werther versus Papageno effects. Br. J. Psychiatry 197, 234–243. doi: 10.1192/bjp.bp.109.074633, 20807970

[ref8] Phillips D. P. (1974). The influence of suggestion on suicide: substantive and theoretical implications of the Werther effect. Am. Sociol. Rev. 39, 340–354. doi: 10.2307/2094294, 11630757

[ref9] Shneidman E. S. (1981). The Psychological Autopsy. Suicide Prevention Center.

[ref10] Yargıtay 12th Criminal Chamber (2015) Equivocal Death Investigation and Psychological Evaluation Principles in Forensic Assessment [Court Decision]. Ankara, Türkiye: Court of Cassation (Yargıtay).

